# Single Molecule Molecular Inversion Probes for High Throughput Germline Screenings in Dystonia

**DOI:** 10.3389/fneur.2019.01332

**Published:** 2019-12-18

**Authors:** Michaela Pogoda, Franz-Joachim Hilke, Ebba Lohmann, Marc Sturm, Florian Lenz, Jakob Matthes, Francesc Muyas, Stephan Ossowski, Alexander Hoischen, Ulrike Faust, Ilnaz Sepahi, Nicolas Casadei, Sven Poths, Olaf Riess, Christopher Schroeder, Kathrin Grundmann

**Affiliations:** ^1^Institute of Medical Genetics and Applied Genomics, University of Tübingen, Tübingen, Germany; ^2^Department of Dermatology, Venereology and Allergology, Charité - Universitätsmedizin Berlin, Berlin, Germany; ^3^Behavioral Neurology and Movement Disorders Unit, Department of Neurology, Istanbul Faculty of Medicine, Istanbul University, Istanbul, Turkey; ^4^Department of Neurodegenerative Diseases, Hertie Institute for Clinical Brain Research, University of Tübingen, Tübingen, Germany; ^5^German Center for Neurodegenerative Diseases, Tübingen, Germany; ^6^Bioinformatics and Genomics Program, Centre for Genomic Regulation, The Barcelona Institute of Science and Technology, Barcelona, Spain; ^7^Universitat Pompeu Fabra, Barcelona, Spain; ^8^Department of Human Genetics, Radboud University Nijmegen Medical Center, Nijmegen, Netherlands; ^9^Radboud Institute of Molecular Life Sciences, Radboud University Nijmegen Medical Center, Nijmegen, Netherlands; ^10^DFG NGS Competence Center Tübingen, Tübingen, Germany

**Keywords:** dystonia, *ATM*, NGS, ataxia-telangiectasia, MIPs

## Abstract

**Background:** This study's aim was to investigate a large cohort of dystonia patients for pathogenic and rare variants in the *ATM* gene, making use of a new, cost-efficient enrichment technology for NGS-based screening.

**Methods:** Single molecule Molecular Inversion Probes (smMIPs) were used for targeted enrichment and sequencing of all protein coding exons and exon-intron boundaries of the *ATM* gene in 373 dystonia patients and six positive controls with known *ATM* variants. Additionally, a rare-variant association study was performed.

**Results:** One patient (0.3%) was compound heterozygous and 21 others were carriers of variants of unknown significance (VUS) in the *ATM* gene. Although mutations in sporadic dystonia patients are not common, exclusion of pathogenic variants is crucial to recognize a potential tumor predisposition syndrome. SmMIPs produced similar results as routinely used NGS-based approaches.

**Conclusion:** Our results underline the importance of implementing *ATM* in the routine genetic testing of dystonia patients and confirm the reliability of smMIPs and their usability for germline screenings in rare neurodegenerative conditions.

## Introduction

Ataxia-telangiectasia (A-T) is a rare autosomal recessively inherited disease usually characterized by ataxia, neuro-motor impairment, ocular or cutaneous telangiectasia, high risk of malignancies and immunodeficiency (1–3). A-T patients are often extraordinarily sensitive to ionizing radiation, contraindicating radiation therapy for them as a standard therapy in case of cancer (4, 5). The disease is caused by mutations of the Ataxia telangiectasia mutated (*ATM*) gene located on chromosome 11q22-23 (6). *ATM* encodes the 350 kDa ATM protein, a nuclear serine/threonine-protein kinase which is crucial in the cellular response to DNA damage such as double-strand breaks (7, 8). Classic A-T is caused by biallelic truncating *ATM* mutations which lead to a total loss of ATM protein, resulting in an impaired cell cycle (9). Since variants in *ATM* are known to confer cancer risk in heterozygous carriers (10), and at the same time cause increased sensitivity of the patients to toxic effects of ionizing radiation (4, 5), identifying *ATM* mutation carriers can be highly relevant for adequate treatment and regular cancer control examinations.

Furthermore, recent reports describe non-classic forms of A-T or “variant A-T” (11, 12). In these cases, some residual ATM kinase function is maintained and the phenotype is highly variable, including incomplete or atypical phenotypes, e.g., ataxia plus extrapyramidal symptoms or choreoathetosis lacking the classical hallmarks, often masking the correct diagnosis (11, 13). Some of these *ATM* mutations manifest as pure generalized or focal dystonia (14).

However, the frequency of *ATM* mutations in different cohorts of dystonia patients is not well-described. Therefore, we tested a cohort of 373 dystonia patients for *ATM* alterations.

We applied single molecule Molecular Inversion Probes (smMIPs) for targeted enrichment and sequencing of all protein coding exons and exon-intron boundaries of *ATM*. SmMIPs represent a cost-efficient and fast high-throughput technique to identify sequence variation in genes containing many exons (15). SmMIPs are oligonucleotide probes possessing two sequences complementary to defined genomic target regions (16–21). These complementary sequences are located at the 3′-end and at the 5′-end of the probe and hybridize to the single-stranded sample DNA upstream and downstream of the chosen target region of variable length. The gap between the complementary sequences is filled with the copy of the DNA target region by a polymerase, circularizing the probe in an additional ligation reaction.

The circular smMIP-target molecule can then be amplified in a PCR reaction and is, after a single library purification step, ready for sequencing. One main advantage of smMIPs is that probes can be designed in a modular way, tiling all relevant regions as closely as necessary, covering both DNA strands if desired (22). This is especially relevant for the avoidance of artifactual DNA sample damage, since artifacts usually only occur randomly in one strand (17, 23). Another asset of smMIPs is the usage of a unique single molecule (sm) molecular identifier (UMI = unique molecular identifier), a sequence of (in our case) 8 random bases in the probe that is individual for 4^8^ ≈ 65,500 molecules. Since every smMIP molecule with an individual UMI-tag can only hybridize to one genomic DNA fragment, the UMI sequence can be used to retrace all originally different DNA molecules and to correct for PCR duplicates in the bioinformatical analysis. Thus, an accurate representation of the diversity of DNA molecules in the sample allows the sensitive detection of variants, even at low frequencies (17, 23, 24). Therefore, it is also most suitable for especially cost-efficient, reliable germline mutation screenings in large cohorts. An asset of the technology is that smMIPs can be designed in a customized way (22) and can be used for massively parallel resequencing of many thousands of target regions (25). In addition, most of the chemistry is independent of a specific supplier.

## Methods

Informed consent was obtained from all patients. All samples were taken in accordance with the local Ethical Committee (# 847/2017BO). Genomic DNA was isolated from blood of 373 dystonia patients of Caucasian origin, who had been examined by specialists in movement disorders according to the current clinical criteria (26). Inclusion criteria were as follows: various degrees of dystonia as defined by published clinical criteria and a clinical course compatible with primary dystonia without features indicating secondary dystonia.

### Probe Design and Pooling

The whole smMIPs protocol was only slightly modified according to established protocols (beside the protocol, a detailed scheme of the methodology and workflow can be found in 15, 17, 21).

We designed smMIPs to screen the patients' DNA using the open source tool MIPGen (22). The design resulted in 190 smMIPs spanning all 62 protein coding exons of the *ATM* gene [transcript ENST00000278616, Ensemble (27)], covering both the sense and anti-sense strand of the DNA. Probes were synthesized by IDT (Integrated DNA Technologies; Coralville, USA). Upon arrival, all smMIPs were pooled in an equimolar manner. Six random samples were processed and sequenced to assess probe performance. Using the read depth of all individual probes, the pool was rebalanced twice, in order to improve uniformity of coverage of the target regions (17, 28) (see Supplementary Material for further information).

### Targeted Enrichment and Amplification

One hundred nanogram isolated DNA were used as input for the targeted capture of genomic regions of interest, and incubated for 21 h with smMIPs, using a ratio of 1:800 (DNA molecules:smMIPs), adding a polymerase and ligase for gap-filling and circularization. Subsequently, a 1-h exonuclease digestion was performed to remove any linear DNA. Thus, only circular smMIPs were amplified in a PCR using a high-fidelity polymerase. A small amount of each PCR product was categorized semi-quantitatively on an agarose gel, and the remaining PCR products were pooled accordingly to obtain equimolar representation of all samples. The pools were cleaned up for Illumina sequencing in one step using XP Ampure beads and the DNA concentration and fragment size was measured using a Qubit 2.0 device and an Agilent TapeStation 2200 to calculate the molarity of each pool. Samples were sequenced on a HiSeq2500 platform with 2 × 125 cycles (paired-end) and a target of 500 clusters per smMIP per sample.

### Sequencing Analyses and Statistics

Sequencing data were analyzed using an adapted in-house pipeline (available on https://github.com/imgag/megSAP, version 0.1-663-ged5a95d). Briefly, all sequences were identified by the UMI to allow later grouping and correction for PCR duplicates. Reads were aligned and mapped using paired-end reads (29), PCR duplicates were used to correct random PCR or sequencing errors labeling their base-quality to 0 to avoid calling falls-positive variants. BAM-files were generated, and variants called using the tool FreeBayes, version 1.1.0 (30). Variants were annotated and saved in GSvar-format.

For rare-variant association, a case-control study was performed (31). As a control cohort, vcf-files of the 1000 genomes project (32) were downloaded for 404 patients with European descent (populations Italian, Spanish, British, and Utah with European ancestry; Finnish not included). In order to obtain a representative and unbiased control cohort, we chose a public database for control data. Only target regions that were covered in both groups, case and control, were analyzed. All variants were subsequently filtered by the following criteria.

For filtering the variants, allele frequencies in the 1000 genomes database and in the ExAC database were necessitated to be below 1% and all variants needed to be reported <50 times in our in-house database. Synonymous and intronic variants (cut-off ± 8 bp) were excluded as well as variants that had been classified as benign or likely benign in our diagnostic in-house database. All variants were classified according to slightly modified guidelines (33, 34).

Statistical analyses were performed using JMP software version 13.0.0 (SAS Institute, Cary, NC). For rare-variant association, a Fisher's exact test was performed (α = 0.05).

The raw data supporting the conclusions of this manuscript will be made available by the authors, without undue reservation, to any qualified researcher. The data (vcf-files) of the control cohort were obtained from the 1000 genomes project (32) and are publicly available.

## Results

The median target region read depth for all samples (*n* = 373) was 218×, ranging from 34× for one sample to 840× in the sample with the highest coverage. The 20×-coverage of all target regions was 98.0%. Note that these figures only comprise condensed reads (no PCR duplicates) which were shown to be sufficient for allele frequencies typical in germline analyses (17).

Among 373 dystonia patients, we found 21 different variants in 22 of the dystonia patients (Table 1). Of these, 20 were of uncertain clinical significance (VUS) (33). Ten of them had high *in silico* prediction of pathogenicity [Combined Annotation-Dependent Depletion, CADD-Score > 20, (35, 36)]. One variant was classified as pathogenic (p. (Val2716Ala), class 5). There was no significant difference from the control group from the 1000 genomes project (*p* > 0.05 for both VUS or predicted pathogenic variants).

**Table 1:** Rare variants in the *ATM* gene among the 373 dystonia patients detected with smMIPs.

**Patient**	**Genomic variant**	**Protein change**	**CADD**	**Classification**
Dys1	c.115A>G	p. (Thr39Ala)	14.38	VUS
Dys2	c.670A>G	p. (Lys224Glu)	23.5	VUS
Dys3	c.670A>G	p. (Lys224Glu)	23.5	VUS
Dys4	c.1066-6T>G	–	–	VUS
	c.7618G>A	p. (Val2540Ile)	20.6	VUS
Dys5	c.1066-6T>G	–	–	VUS
Dys6	c.1066-6T>G	–	–	VUS
Dys7	c.2386A>T	p. (Asn796Tyr)	7.75	VUS
Dys8	c.3802G>A	p. (Val1268Met)	26.9	VUS
Dys9	c.4060C>A	p. (Pro1354Thr)	14.83	VUS
Dys10	c.4297T>G	p. (Tyr1433Asp)	27.3	VUS
Dys11	c.4388T>G	p. (Phe1463Cys)	28.7	VUS
Dys12	c.4424A>G	p. (Tyr1475Cys)	18.12	VUS
Dys13	c.5005+7_5005+8delTA	–	–	VUS
Dys14	c.5693G>A	p. (Arg1898Gln)	18.17	VUS
Dys15	c.5890A>G	p. (Lys1964Glu)	17.81	VUS
Dys16	c.6860G>C	p. (Gly2287Ala)	10.23	VUS
Dys17	c.6983C>T	p. (Pro2328Leu)	22.4	VUS
Dys18	c.7276C>T	p. (Leu2426Phe)	29.3	VUS
Dys19	c.7475T>G	p. (Leu2492Arg)	28.3	VUS
Dys20	c.7796C>T	p. (Thr2599Ile)	11.78	VUS
Dys21	c.8147T>C	p. (Val2716Ala)	26.2	5
	c.8578_8580delTCT	p. (Ser2860del)	–	VUS^*^
Dys22	c.8314G>A	p. (Gly2772Arg)	31	VUS

*CADD-scores (Combined Annotation-Dependent Depletion) (35, 36) are not available for intronic variants and deletions*.

* *Evidence in literature for pathogenicity (14), but missing functional analyses*.

Among the dystonia patients, one patient was found with two variants in the *ATM* gene: one was pathogenic and one a VUS with evidence for pathogenicity (p. (Val2617Ala), p. (Ser2860del)). Clinical examination had produced highly elevated AFP levels, familial aggregation of malignancies and a deterioration of the dystonic symptoms that were largely insensitive to treatment. This patient was recently published (14). In contrast, we found no biallelic pathogenic or likely pathogenic variants in the 1000 genomes control cohort.

All pathogenic variants reported for the **six** positive controls were also detected using smMIPs. The comparison of all variants showed no discrepancy between smMIPs and the diagnostic results.

## Discussion

### Dystonia

Although we found no statistical association of *ATM* variants between dystonia patients and a control group, we found one patient with a pathogenic variant (p. (Val2617Ala)) and a VUS with evidence for pathogenicity (p. (Ser2860del)) among 373 dystonia patients. In accordance with other published data on the patient (14), our study confirms the efficiency of smMIPs as a diagnostic screening tool (15) and moreover the importance of identifying *ATM* as rare underlying cause in dystonia (37, 38). The genetic causes of dystonia, a rare disease, are very heterogeneous (39), and many genes have been identified in recent times. Among these genetic causes, *ATM* sequence variations are a crucial factor for patients regarding cancer susceptibility (10), disease progression and radiation toxicity (3, 4, 40, 41). Thus, we stress the importance of including *ATM* to the general screening for causes of dystonia.

### smMIPs

Due to the usage of UMIs, smMIPs increase the confidence in variants without the necessity of a high coverage, because PCR duplicates can be used to correct for artifacts (17, 23). We successfully established smMIPs in a screening for *ATM* germline variants in dystonia patients. All pathogenic variants that had been detected with different diagnostic NGS approaches in six control patients (and one dystonia patient) were confirmed, validating smMIPs as a sensitive, competitive methodology. They provide a straight-forward wet lab protocol, with customizable chemistry, flexible probe design, sensitive variant detection, and cost-efficient sample processing, especially for high-throughput genetic testing (15, 21, 28, 42). Thus, they constitute a convenient tool for panel-based genetic testing.

## Conclusion

Since *ATM* mutations confer a higher risk of developing cancer and radiation toxicity, it is crucial to detect *ATM*-variants as the underlying cause in any dystonic patient. In our study, we found smMIPs to be as sensitive as other NGS-based approaches while being highly cost-efficient and flexible.

## Data Availability Statement

The raw data supporting the conclusions of this article will be made available by the authors, without undue reservation, to any qualified researcher. The data (vcf-files) of the control cohort were obtained from the 1000 genomes project and are publicly accessible here: http://www.internationalgenome.org/data#download.

## Ethics Statement

All experimental protocols in this study were reviewed and approved by the Ethics Committee of the Eberhard Karls University of Tübingen and of the Medical Faculty of the University Hospital Tübingen. All patients gave written informed consent in accordance with the Declaration of Helsinki. The patients/participants provided their written informed consent to participate in this study.

## Author Contributions

The research project was conceptualized by OR, CS, F-JH, EL, and KG. Sample acquisition was accomplished by EL, KG, and UF. Methodology was implemented and supported by AH, F-JH, MP, NC, and SP. The computational analyses were developed and/or conducted by CS, MS, FL, SO, FM, and JM. Variant classification and (statistical) analyses were conducted by MP, IS, and SO. All authors revised and approved the final manuscript.

### Conflict of Interest

The authors declare that the research was conducted in the absence of any commercial or financial relationships that could be construed as a potential conflict of interest.
